# *Mannheimia haemolytica* serovars associated with respiratory disease in cattle in Great Britain

**DOI:** 10.1186/s12917-021-03121-3

**Published:** 2022-01-03

**Authors:** Colin Mason, Jane Errington, Geoffrey Foster, Jennifer Thacker, Oliver Grace, Katharine Baxter-Smith

**Affiliations:** 1 SRUC Veterinary Services, St Marys Industrial Estate, Dumfries, DG1 1DX UK; 2 SRUC Veterinary Services, An Lochran, 10 Inverness Campus, Inverness, IV2 5NA UK; 3 SRUC Veterinary Services, Pentlands Science Park, Bush Loan, Penicuik, Midlothian, EH260PZ UK; 4 MSD Animal Health, Milton Keynes, MK77AJ UK

**Keywords:** *Mannheimia haemolytica*, Serotypes, Bovine, Respiratory disease, Pneumonia, Serovars, Cattle

## Abstract

**Background:**

*Mannheimia haemolytica* is commonly associated with respiratory disease in cattle worldwide as a cause of fibrinous pneumonia, bronchopneumonia and pleuritis. *M. haemolytica* is further subdivided into 12 serovars, however not all are considered to be pathogenic in cattle. The study aim was to determine the most common serovars of *M. haemolytica* associated with respiratory disease in cattle in Great Britain, which is currently unknown and could be useful information for clinicians when considering preventative strategies.

**Results:**

One hundred four *M. haemolytica* isolates isolated from bovine clinical pathology and post-mortem samples from pneumonia cases between 2016 and 2018 were tested using a multiplex PCR assay to identify *M. haemolytica* serovars A1, A2 and A6. 46 isolates (44.2%) typed as *M. haemolytica* serovar A1, 31 (29.8%) as *M. haemolytica* serovar A2 and 18 isolates (17.3%) as *M. haemolytica* serovar A6. Nine isolates (8.7%) were not A1, A2 or A6 so were considered to belong to other serovars or were not typable.

**Conclusion:**

This study highlights the importance of *M. haemolytica* serovars other than A1 which may be responsible for respiratory disease in cattle and could help guide the veterinarian when making choices on preventative vaccination programmes.

**Supplementary Information:**

The online version contains supplementary material available at 10.1186/s12917-021-03121-3.

## Background

*Mannheimia haemolytica* is associated with respiratory disease in cattle and sheep worldwide. The organism can be a primary component of pneumonia secondary to environmental stressors as well as infecting cattle synergistically with other bacterial, viral and Mycoplasma species [1].

There are twelve serovars of *M. haemolytica*, based on capsular antigens and labelled A1, A2, A5, A6, A7, A8, A9, A12, A13, A14, A16 and A17. A further serovar, A11, is restricted to *M. glucosida* [2, 3].

*M. haemolytica* serovars A1, A2 and A6 are the most prevalent worldwide [4] and are readily isolated from the nasopharynx of healthy cattle. Serovars A1 and A6 have been reported as common isolates from pneumonic lung tissue [5], serovar A2 is a major cause of disease in sheep [6] and has traditionally been considered as a commensal organism in cattle; however, a recent review paper highlighted some evidence to suggest this serovar may not be purely commensal in this species [7].

Several vaccines are licenced in Great Britain for protection against *M. haemolytica* but not all vaccines cover serovars A1 and A6. Reviews of the common serovars of *M. haemolytica* associated with respiratory disease can inform pharmaceutical companies on serovars required for inclusion in vaccines and to help veterinary surgeons make informed decisions on vaccine choice.

A multiplex PCR assay can provide a rapid, simple and cost-effective alternative to slide agglutination assays currently used for *M. haemolytica* serotyping and avoids the need to use laboratory animals for serotyping assays. We validated a PCR test for the most prevalent serovars of *M. haemolytica* in cattle based on the test development and primers described by Klima et al. [8]. We used this test to investigate the prevalence of each of these serovars amongst *M. haemolytica* isolates cultured from diagnostic material from bovine respiratory disease submitted to SRUC Veterinary Service laboratories in the years 2016–2018.

## Results

The results of specificity testing of the *M. haemolytica* PCR assay confirmed that all *M. haemolytica* serovar A1, A2 and A6 strains were correctly identified. All confirmed non-*M. haemolytica* strains were negative using the *M. haemolytica* PCR assay.

The largest proportion of isolates (44.2%) typed as *M. haemolytica* serovar A1, 29.8% typed as *M. haemolytica* serovar A2 and 17.3% typed as A6. Nine isolates (8.7%) were not A1, A2 or A6 so were considered to belong to other serovars or were not typable (Table 1).

**Table 1:** Typing results for 104 UK *Mannheimia haemolytica* isolates

	***Mannheimia haemolytica*** serovar A1	***Mannheimia haemolytica*** serovar A2	***Mannheimia haemolytica*** serovar A6	***Mannheimia haemolytica*** (other than A1, A2 or A6)
**Number of isolates (%)**	46 (44.2%)	31 (29.8%)	18 (17.3%)	9 (8.7%)

## Discussion

The work presented here demonstrates that a multiplex PCR assay incorporating three serotype specific primer pairs for identification of *M. haemolytica* serovars A1, A2 and A6 is an effective test for typing isolates of these serovars. The test validation against known serovars and isolates showed that all confirmed *M. haemolytica* serovar A1, A2 and A6 strains were correctly identified using the *M. haemolytica* PCR assay. All confirmed other strains of *M. haemolytica* were negative using the *M. haemolytica* PCR assay.

The 104 *M. haemolytica* isolates tested were selected through scanning surveillance which does not allow general population conclusions to be drawn from any sample set that is collected in this way. One other source of bias was that a single colony isolate was selected from each case. However, the testing does confirm that *M. haemolytica* serovar A1 was identified most commonly and serovar A6 least commonly. A more structured survey would be required to estimate the relative prevalence of these different strains generally in the cattle population and in cases of respiratory disease.

The finding that *M. haemolytica* serovar A1 is the most common serovar in this set of British isolates is consistent with other studies of European isolates [9]. A large proportion of respiratory samples typed as serovar A2, moreover 97% of the *M. haemolytica* serovar A2 isolates were isolated from pneumonic lung tissue with gross pathology consistent with bacterial pneumonia. Histopathology was carried out on 40% of the cases and this detailed examination confirmed lesions consistent with bacterial bronchopneumonia. This serovar has previously been reported as a commensal organism in North America, however a more recent review paper stated, ‘the commonly accepted idea that *M. haemolytica* A2 isolates are primarily non-pathogenic bacteria associated with healthy cattle should be considered with caution’ [7]. Therefore, more research is required to understand the contribution of this serovar to the bovine respiratory disease complex.

Not all vaccines against *M. haemolytica* cover all the major serovars and studies of this nature may be of assistance in the selection of the appropriate bovine respiratory disease vaccination programme.

## Conclusion

*M. haemolytica* serovars A1 and A6 were commonly isolated from cases of bovine respiratory disease in this study, mirroring similar research in other countries. A large proportion of clinical samples typed as serovar A2, however more work is required to fully understand the contribution of this serovar to the bovine respiratory disease complex. These results could help veterinarians select the appropriate preventative vaccination programme when pneumonia attributed to *M. haemolytica* is diagnosed on UK cattle farms.

## Methods

A multiplex PCR was validated for this study using three serotype specific primer pairs for identification of *M. haemolytica* serovars A1, A2 and A6 based on published work [8]. DNA was extracted and amplified using standard techniques and the serotype specific primers. PCR amplification was performed using a conventional thermal cycler and reaction products were separated by electrophoresis on a 2% agarose gel. Images of the gel were captured and analysed on an Image Lab 4.0 Image Analysis system. Negative and positive controls were included in each run with the positive controls comprising DNA extracts of each *M. haemolytica* serovar 1, 2 and 6. Identification of *M. haemolytica* serovars in clinical isolates was determined by comparing PCR amplification product band sizes with those in the positive PCR control lanes (*M. haemolytica* serovars A1, A2 and A6) of the analysed gel.

Specificity of the *M. haemolytica* PCR assay was determined by testing panels of known *M. haemolytica* serovars (A1, A2 and A6), un-typed *M. haemolytica* strains and closely related strains including *Bibersteinia trehalosi, Pasteurella multocida, Mannheimia glucosida, Mannheimia varigena* and *Mannheimia granulomatis.*

The sample set comprised material received from one hundred and four cases or disease outbreaks with a clinical history of respiratory disease occurring between 2016 and 2018, each defined as a submission. All submissions produced an isolate of *M. haemolytica* and only one isolate per submission and per affected farm was included in the study. Submission selection was not random but was selected as a representative sample from the overall number of submissions of respiratory disease cases to the SRUC laboratories between 2016 and 2018 to give as wide a UK distribution as possible and be representative of farm type (dairy or beef), animal breed, age, and sex.

The sample set included a mixture of breeds and cross-bred animals derived from 70 animals on beef units, 25 animals on dairy units and no data was available for 9 samples. Where the gender of the animal was recorded, 41 animals were female and 41 were male. The age range of the animals from which samples were derived was from 1 day to 8 years old with 46 animals 0–3 months old, 19 animals > 3 months and < 6 months old, 16 animals 6 months – 2 years old, 5 animals > 2 years old and 18 animals where the age was not available. Results were obtained from animals throughout the Great Britain, 22 from England, 4 from Wales and 78 from Scotland.

Sample type comprised nasopharyngeal swabs (17 cases), broncho-alveolar lavage fluid (2 cases) submitted by veterinary practitioners and post-mortem material (85 cases). Bovine carcases submitted to SRUC Veterinary Services post-mortem rooms by livestock keepers were examined for the presence of visible lesions of pneumonia and affected lung was then sampled for bacterial culture. For all sample types, primary bacterial cultures were made on Columbia sheep blood agar (CSBA) (Thermo-Fisher, Perth, UK), following 18–24 h incubation at 37 °C in capnophilic conditions. Identification was confirmed using routine phenotypic tests and isolates were stored at − 80 °C in Microbank® cryovials (Prolab Diagnostics, Wirral, UK). The pathogenic significance of the isolates was assessed by clinical history and the pathology seen thus providing an accurate representation of 104 *M. haemolytica* isolates of clinical significance.

*M. haemolytica* isolates derived from the above submissions were revived by culture on CSBA for testing in the PCR assay.

## Supplementary Information

**Additional file 1.**

## Data Availability

The datasets used and/or analysed during the current study are available from the corresponding author on reasonable request.

## Abbreviations

*M. haemolytica*: *Mannheimia Haemolytica*
PCR: Polymerase Chain Reaction
SRUC: Scottish Rural College
CSBA: Columbia Sheep Blood Agar
